# Breaking the paradox: simultaneous recovery of phosphorescence and mechanical properties in polymeric films

**DOI:** 10.1039/d5sc09282e

**Published:** 2025-12-29

**Authors:** Yan Wang, Kaitao Li, Yongpeng Yang, Rui Tian, Chao Lu

**Affiliations:** a State Key Laboratory of Chemical Resource Engineering, Beijing University of Chemical Technology Beijing 100029 China tianrui@mail.buct.edu.cn luchao@mail.buct.edu.cn; b Pingyuan Laboratory, College of Chemistry, Zhengzhou University Zhengzhou 450001 China; c Quzhou Institute for Innovation in Resource Chemical Engineering Quzhou 324000 China

## Abstract

Polymeric films with self-healable room temperature phosphorescence (RTP) and mechanical performance are eagerly anticipated for wearable and electronic devices. However, simultaneously recovering phosphorescence and mechanical properties remains a great challenge due to improper interactions quenching phosphorescence and the inherent conflict between chain rigidity and flexibility in polymeric films. Herein, we propose the use of a chromophore binder between the polymer matrix to fabricate RTP films with simultaneous recovery of phosphorescence and mechanics. A covalent cross-linking network was established, restricting the molecular motion of chromophore binders to achieve bright deep-blue phosphorescence emissions. Additionally, the films exhibited processability, flexibility, stretchability, and self-healing ability. Both the phosphorescent and mechanical properties could be recovered with an efficiency of more than 90% for the films healed in water under room temperature. Theoretical simulation showed that this noteworthy self-healing capacity could be ascribed to the relatively low energy for the formation and re-formation of the covalent cross-linkage between the chromophore binders and polymer matrix. Accordingly, we realized an assembly–reassembly process for multi-emission phosphorescence by healing film fragments using different chromophores with boronic acid groups. It is anticipated that this facile and universal strategy *via* covalent cross-linkage could provide possibilities for the design of multi-functional optical materials with expanded application fields.

## Abstract 2

We have realized the simultaneous recovery of phosphorescence and mechanical properties for polymeric films based on covalent cross-linking between the chromophore binders and polymer matrix.

## Graphical abstract

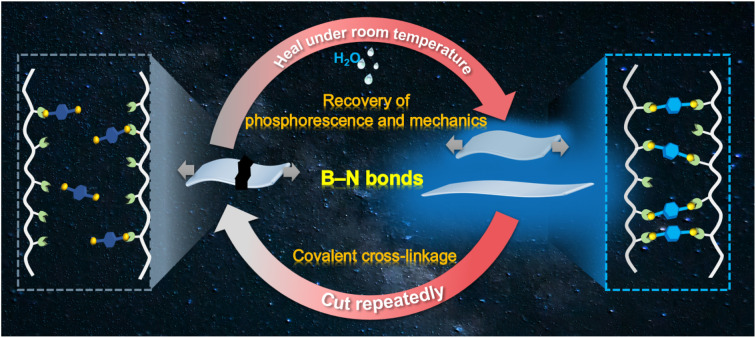

## Introduction

Room-temperature phosphorescence (RTP) films with good mechanical performance have shown great promise in flexible optical communication,^1–3^ displays, and e-skin.^4–6^ These applications impose strict demands in terms of mechanical deformation with sustainable phosphorescence emissions. Unfortunately, overloading with mechanical force may induce structural damage and functional degradation in these RTP films, ultimately leading to a decay in lifetime and loss of encrypted data.^7–9^ Currently, a self-healing strategy to restore structure and functionalities has been proposed,^10–12^ which requires high chain mobility to diffuse along the polymer interfaces for the healing process. However, it is well known that flexible and mobile chains are detrimental to depress the non-radiative transition for phosphorescence emissions, which may lead to the compromise of phosphorescence loss in the self-healed films. Although the mechanical properties could be recovered sometimes, improper healing conditions can quench the triplet excitons and lead to the failure to recover phosphorescence. Therefore, great efforts should be made to solve the conflict between the rigidity and flexibility of polymers for self-healable polymeric films to recover both phosphorescence and mechanics.

The incorporation of a small-molecular binder between the polymer matrix has been proposed as a facile self-healing strategy.^13,14^ Specifically, the interaction between the binder and polymer matrix is vital to recover the functionalities of the polymeric films by changing the functional groups of the binder.^15^ To date, dynamic covalent or non-covalent interactions have been exploited to realize self-healing strategies, such as disulfide bonds,^16^ imine bonds,^17^ Diels–Alder bonds, and borate ester bonds.^18,19^ However, most of these covalent bonds are initiated by external energy. For example, imine and boron ester bonds, which have a bonding energy of 600 and ∼500 kJ mol^−1^,^20^ require heat energy to trigger the reaction for self-healing. Polymers with disulfide bonds could be self-healed under 70 °C,^21^ and Diels–Alder bonds could be formed under 80 °C.^22^ Notably, most phosphorescent chromophores are unstable under thermal treatment, and the heat-intensified non-radiative transition leads to a decayed persistent afterglow and hindered healing efficiency.^23,24^ Alternatively, hydrogen bonds with low binding energy could be adopted to heal polymers at room temperature. Disappointingly, the uptake of water generally occurs in the presence of hydrogen bonds, leading to reduced stiffness and quenched RTP properties in the polymeric films.^25,26^ Therefore, the design of a facile strategy to achieve high self-healing efficiency at room temperature without phosphorescence decay remains a current challenge.

**Scheme 1:**
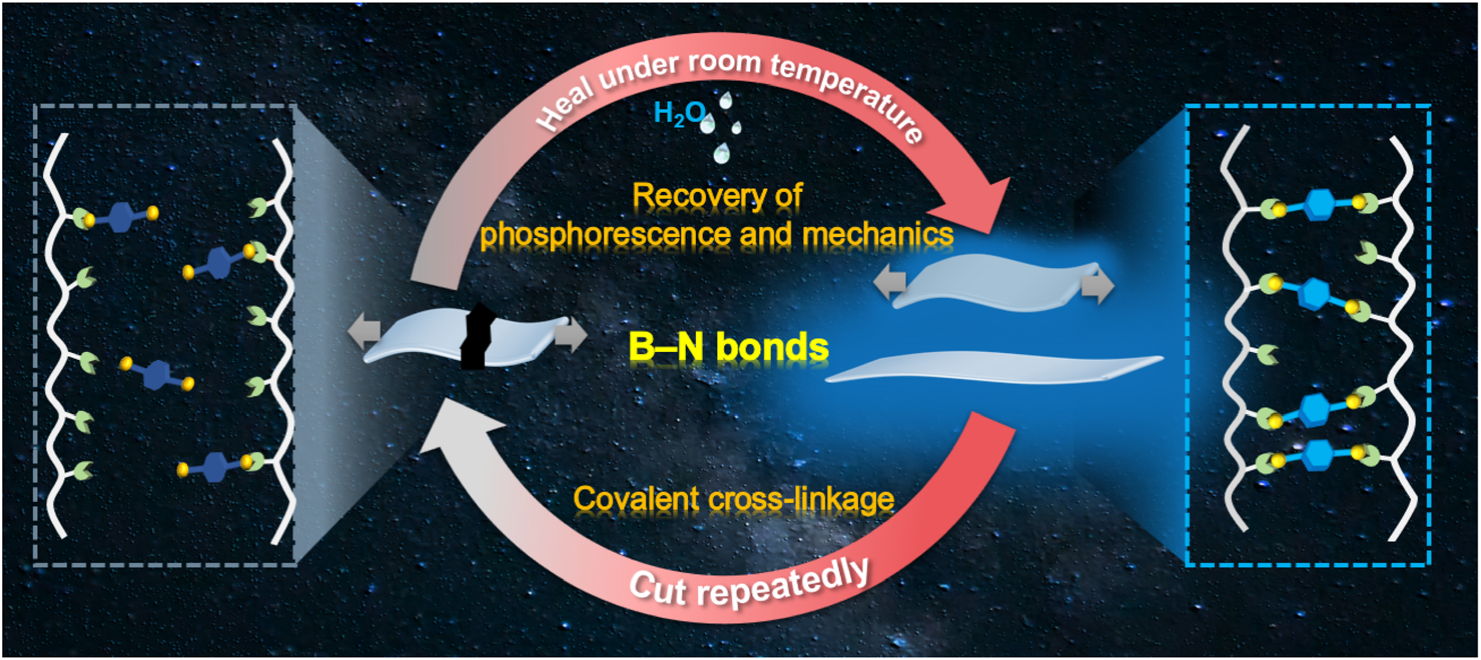
Schematic diagram of the preparation of a self-healing material that exhibits simultaneous recovery of phosphorescent and mechanical performance through B–N covalent bonds.

B–N bonds with a binding energy of around 100 kJ mol^−1^ can be formed under mild conditions through a click reaction.^27^ In this work, we have employed B–N covalent bonds to prepare a self-healable RTP material based on polyacrylamide (PAM) with abundant amino groups and chromophore binders with boronic acid groups (Scheme 1). Bright deep-blue phosphorescence was obtained due to the restricted molecular motion and regulated molecular arrangement of the chromophore binders. The as-prepared polymeric RTP films exhibited an elongation rate of 530% with uniformly distributed phosphorescence upon stretching. Noteworthily, not only the mechanical performance, but also the RTP of the films, could be simultaneously recovered to more than 90% of the pristine values upon self-healing in water at room temperature. This cutting–healing cycle could be repeated consecutively through the breakage and re-formation of covalent bonds. We have confirmed the advantages of facile B–N covalent cross-linkage in promoting RTP and self-healing performance through a condensation energy calculation using a density-functional theory simulation. Furthermore, the recyclability of these self-healing RTP films was validated by assembly–reassembly of different pieces of the cross-linked films. As expected, multiple phosphorescence emissions could be acquired by self-healing, and the healed sites exhibited good phosphorescence and mechanical performance even under folding, stretching and rotation. Therefore, the simultaneous recovery of the deep-blue RTP and mechanical properties was observed for polymeric RTP films, demonstrating the advantages of covalent cross-linkage between chromophore binders and polymer matrices. It is anticipated that the established covalent cross-linking strategy could open a new avenue for the design of practical optical devices with enhanced durability, safety, and security of stored confidential information.

## Results and discussion

### RTP and mechanical properties of the *x*% BDBA-PAM films

**Fig. 1:**
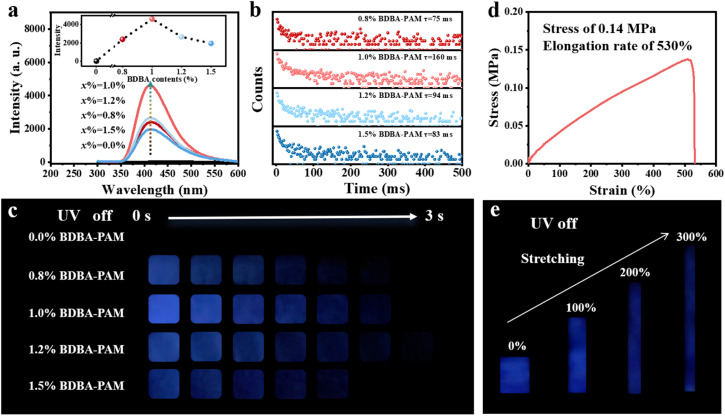
RTP behavior and mechanical performance of the *x*% BDBA-PAM films. (a) phosphorescence emission spectra (inset shows the variations among the RTP intensities); (b) phosphorescence lifetimes and (c) photographs of *x*% BDBA-PAM films; (d) mechanical property curve of the 1% BDBA-PAM composite film and (e) photos of the phosphorescence of the BDBA-PAM films under different stretching rates.

We chose 1,4-benzenediboronic acid (BDBA), which possesses two boronic acid groups attached at the terminals of the phenyl group, as the binder to construct the composite films. Green phosphorescence emission centered at 500 nm was observed for BDBA powder under excitation at 280 nm (Fig. S1 and S2). In comparison, the low-temperature phosphorescence spectra of an aqueous solution of BDBA (10^−6^ mol L^−1^) recorded in liquid nitrogen showed dark-blue emission at around 410 nm (Fig. S3). The two different emission centers at 500 and 410 nm suggested the aggregated and isolated states of BDBA, respectively.^28^ Accordingly, the BDBA molecule with boronic acid groups was selected as the binder to cross-link PAM with amino groups through B–N bonds. BDBA-PAM composite films were successfully prepared, and the elements boron, carbon, oxygen, and nitrogen uniformly distributed throughout the films, as observed from scanning electron microscopy and energy dispersive spectrometry (Fig. S4). All the *x*% BDBA-PAM films showed deep-blue phosphorescence emission at 410 nm under an excitation wavelength of 280 nm, while the pure PAM showed no phosphorescence emission (Fig. 1a). This deep-blue phosphorescence could be ascribed to the depressed self-condensation and isolated states of BDBA molecules after reaction with PAM through the covalent bonds. The RTP emission of the *x*% BDBA-PAM films was promoted with the increase of the BDBA content (*x*%) from 0 to 1.0%, followed by a decrease due to excess BDBA at 1.5% (Fig. 1a, inset). The decay in the RTP performances for 1.5% BDBA-PAM could be ascribed to the fact that the amino groups in the PAM were not sufficient to localize all the BDBA molecules through B–N bonds, and some free BDBA molecules dissipated energy through molecular motion. A similar tendency was also observed for the lifetimes of the *x*% BDBA-PAM films: the lifetimes at 410 nm showed an increase to 160 ms for the 1.0% BDBA-PAM film and then a subsequent decrease (Fig. 1b). The temperature-dependent lifetimes validated the nature of the phosphorescence for the BDBA-PAM composite films (Fig. S5). These phenomena were validated by the photographs of the *x*% BDBA-PAM films using UV-LEDs as a light source. A deep-blue afterglow of around 3 s could be observed by the naked eye for the *x*% BDBA-PAM films when the UV lamp was switched off (Fig. 1c), and the 1.0% BDBA-PAM film showed the strongest phosphorescence intensity and the longest lifetime. In comparison, no phosphorescence could be observed for the pure PAM upon irradiation. These results suggested the enhanced phosphorescence of the BDBA molecules, which could be ascribed to the increased rigidity of the composite films based on the covalent network between BDBA and PAM.

The mechanical properties of the BDBA-PAM composite films were studied *via* tensile testing measurements. The stress–strain curve showed that the stress of the 1.0% BDBA-PAM composite film was 0.14 MPa, and its elongation rate could reach 530% (Fig. 1d). In comparison, the elongation rate of pristine PAM was only 180% (Fig. S6). This comparison demonstrated the good flexibility of the cross-linked BDBA-PAM film. To investigate the RTP performance of the film under stretching, the 1.0% BDBA-PAM film was stretched to 0, 100, 200 and 300% of its original length, and photos of the phosphorescence were captured after turning off the UV lamp. A uniform and strong deep-blue afterglow was observed for the 1.0% BDBA-PAM film at all stretching rates from 0% to 300% (Fig. 1e), which could be ascribed to the uniform distribution of BDBA as a binder to cross-link PAM in the composite films. These results demonstrated the stable and good RTP performance of the BDBA-PAM composite films under different elongation rates.

### Self-healing performance of the *x*% BDBA-PAM films

**Fig. 2:**
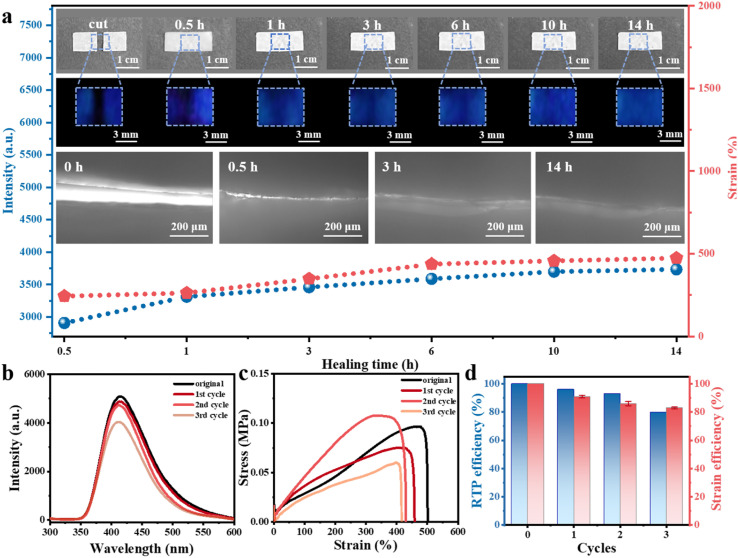
RTP and mechanical performance of the 1% BDBA-PAM film during the room temperature self-healing process using water as the healing reagent. (a) Variation in the phosphorescence and mechanical properties of the BDBA-PAM films at different self-healing times; the insets show photos under daylight after the UV light was turned off and electron microscope images of the BDBA-PAM films during the self-healing process. (b) Phosphorescence spectra, (c) stress–strain measurements and (d) self-healing efficiencies of the BDBA-PAM films after repeated cutting–healing cycles.

The self-healing ability of the BDBA-PAM composite film was studied. In a typical healing experiment, the film was cut into two pieces. Water, an environmentally friendly reagent, was added dropwise to the fracture between these two pieces for healing. Upon the addition of the healing agent (water) at room temperature, the motion of the BDBA and PAM molecules was initiated at the fracture, leading to the reconstruction of B–N covalent bonds at the interfaces. The self-healing process of the BDBA-PAM composite film proceeded at room temperature. After 5 min of treatment, the two pieces of BDBA-PAM film had self-healed and could be picked up as a single piece using a tweezer (Fig. S7a). In contrast, the pure PAM film failed to heal under the same condition (Fig. S7b), suggesting the key role of binders with boronic acid groups in achieving the self-healing ability of the BDBA-PAM composite films. With prolonged treatment time, more B–N covalent bonds were reconstructed to heal the BDBA-PAM composite film. The BDBA-PAM composite films were recovered to their original shape after 0.5 h of treatment, and no cracks could be observed in photos taken under daylight (Fig. 2a, inset, top). The evolution of the fracture in the BDBA-PAM composite film during the healing process could be observed *via* the phosphorescence after turning off the UV light. Faint phosphorescence emissions could be observed near the fracture after self-healing for 0.5 h, and the blue afterglow recovered gradually when the time was prolonged to 3 h. Bright and strong phosphorescence could be observed throughout the film after 14 h of self-healing (Fig. 2a, inset, top). The self-healing ability of the BDBA-PAM composite film was further observed using an electron microscope. The cracks grew smaller as the healing time was prolonged to 3 h and were healed after 14 h (Fig. 2a, inset, bottom). These results demonstrated the recovery of the completeness and phosphorescence of the BDBA-PAM composite films after the self-healing process. For comparison, we prepared reference film samples employing PVA as the polymer matrix. The hydroxyl groups in PVA could react with BDBA molecules to form B–O covalent bonds. However, the cut pieces of BDBA-PVA film could not be healed using the same cutting-healing process, and no phosphorescence emission was observed for these films (Fig. S8). These results demonstrated the advantages of B–N covalent bonds over B–O bonds for preparing cross-linked BDBA-PAM composite films, which could be self-healed under room temperature using water as the healing agent.

Both RTP and mechanical properties were used as key parameters to evaluate the self-healing efficiencies of the BDBA-PAM composite films. The phosphorescence intensity at the fracture recovered to 71% after 0.5 h of healing (Fig. S9a), followed by a continuous increase to 92% at 14 h (Fig. 2a and Table S1). Moreover, the mechanical properties of the BDBA-PAM composite films also showed an increasing trend: the elongation rate reached 478% after 14 h of healing (Fig. S9b), which was more than 90% of the value of the pristine film (Table S1). This cutting–healing process was then repeated for the BDBA-PAM film under room temperature using water as the healing reagent. Satisfactorily, the phosphorescence emission of the BDBA-PAM film recovered to 90% of the original intensity after three consecutive cutting–healing cycles (Fig. 2b and Table S2), and the healing efficiency of the mechanical properties could reach 83% (Fig. 2c and d). Therefore, efficient self-healing performance was realized for the BDBA-PAM films, with recovery of both the phosphorescence and mechanical behaviors.

### Structural characterization of the *x*% BDBA-PAM films during the self-healing process

**Fig. 3:**
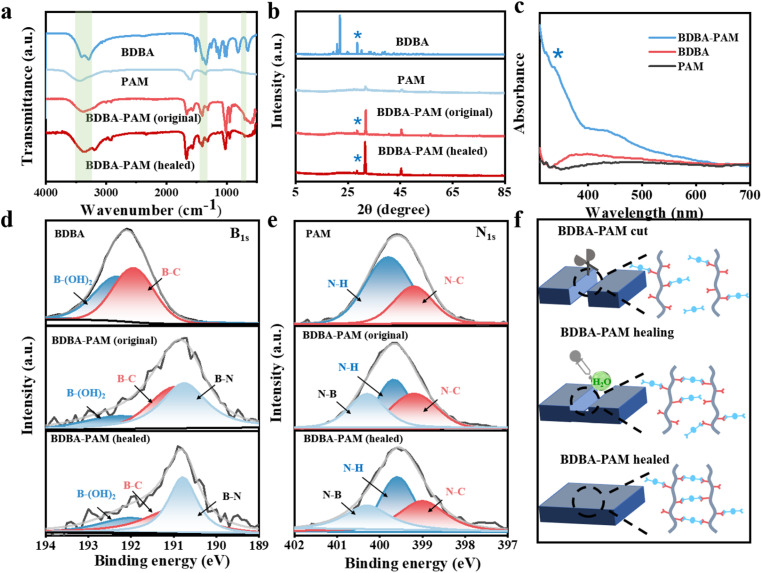
Structural studies of *x*% BDBA-PAM composite films before and after the self-healing process. (a) FT-IR spectra, (b) XRD patterns, (c) UV-Vis spectra, and (d) B 1 s and (e) N 1s spectra of BDBA, PAM, BDBA-PAM films before and after the healing process. (f) Mechanism diagram of the healing process of BDBA-PAM based on B–N covalent cross-linking.

In order to investigate the interaction between BDBA and PAM, Fourier transform infrared spectroscopy (FT-IR) was carried out on the BDBA-PAM composite films.^29,30^ The absorption peaks of the O–H bonds in the range of 3200–3400 cm^−1^ and the characteristic peak of the B–O bond near 1340 cm^−1^ were observed for BDBA (Fig. 3a). PAM showed characteristic peaks around 3445 cm^−1^ attributed to the stretching vibration of NH_2_.^31^ This characteristic peak of PAM shifted to lower wavenumber after reaction with BDBA, indicating the decreased content of amino groups due to the formation of covalent B–N cross-linking. Notably, new peaks at 1398 cm^−1^ and 720 cm^−1^ were identified in the BDBA-PAM composite film, which were ascribed to the stretching vibration and bending vibration of the B–N bonds.^32^ The appearance of these peaks verified the successful covalent cross-linking between BDBA and PAM in the composite film. Notably, these peaks ascribed to the B–N bond were be identified in the BDBA-PAM film healed in water, suggesting the successful reconstruction of B–N bonds in the film after the healing process.

X-ray diffraction (XRD) measurements were further implemented for the BDBA-PAM films. The characteristic diffraction peaks of PAM appeared at 31.9° and 45.4°, indicating the crystallization of PAM (Fig. 3b). After reaction with BDBA, the characteristic peak of BDBA at 28.5° was identified in the BDBA-PAM composite films. In addition, the crystalline peaks of PAM at 31.9° and 45.4° increased significantly from 11.59% to 30.26%, suggesting improved molecular chain regularity and enhanced crystallinity of PAM in the BDBA-PAM composite films. This phenomenon could be ascribed to the modification of the molecular arrangement of PAM after the covalent cross-linking reaction with the BDBA binder.^33^ After the cutting–healing process, the PAM in the BDBA-PAM composite showed a similar peak shape with a crystallinity of 34.22%, suggesting that PAM retained the same structure after the breakage and reconstruction of the B–N bonds.

The UV-Vis absorption spectra of BDBA, PAM and the BDBA-PAM composites were further studied. A characteristic *n*–π* transition with absorption peaks at 250–400 nm^34^ was identified, and was significantly enhanced for the BDBA-PAM composite film compared to that of BDBA (Fig. 3c). This result demonstrated that the N atoms in PAM could provide lone-pair electrons towards the benzene unit through the covalent B–N bonds and facilitate the *n*–π* transition for phosphorescence in BDBA-PAM.

The changes in chemical bonding in the composite films were further investigated using X-ray photoelectron spectroscopy (XPS).^35,36^ The B 1 s peak of BDBA at 192.08 eV could be split into B–(OH)_2_ (192.30 eV) and B–C (191.90 eV) peaks.^37^ The B 1 s peak shifted to 190.80 eV in the BDBA-PAM composite films (Fig. 3d), with the appearance of new peaks for B–N (190.70 eV). Note that the content of B–(OH)_2_ decreased from 50.0% in BDBA to 16.2% in the BDBA-PAM composite films, with the accumulated content of B–N rising to 38.6% (Table S3). After the healing process, the content of B–N bonds was about 37.1%, suggesting the successful reconstruction of the BDBA-PAM composite films. In the N 1s spectra, N–H (399.80 eV) and N–C (399.19 eV) bimodal peaks were observed for PAM,^38^ and a new peak ascribed to N–B was observed at 400.20 eV in the BDBA-PAM composite film (Fig. 3e). The N–H bonds decreased from 66.7% in PAM to 33.5% in BDBA-PAM composite, which was associated with the increased content (33.3%) of N–B (Table S4). These results indicated that the covalent interactions consumed the NH_2_ in PAM, and that N–B bonds were formed in the composite films. After the healing process, we identified the reconstruction of N–B bonds from their almost unchanged content before and after the healing process. Similar changes were also observed in the C 1s spectra of the BDBA, PAM and BDBA-PAM composite films (Fig. S10 and Table S5), based on the C–C/C=C bond peak in the C 1s spectrum at 284.8 eV.^39^ These results demonstrated the formation of covalent cross-linking between BDBA and PAM in the composite films, enhancing the network rigidity and promoting RTP/mechanical properties. The dynamic reversibility of these B–N covalent bonds endowed the composite materials with self-healing ability, *i.e.*, they were broken in the cutting process and restored *via* healing in water (Fig. 3f).

### Theoretical simulation of the phosphorescence and self-healing mechanisms for the composites

**Fig. 4:**
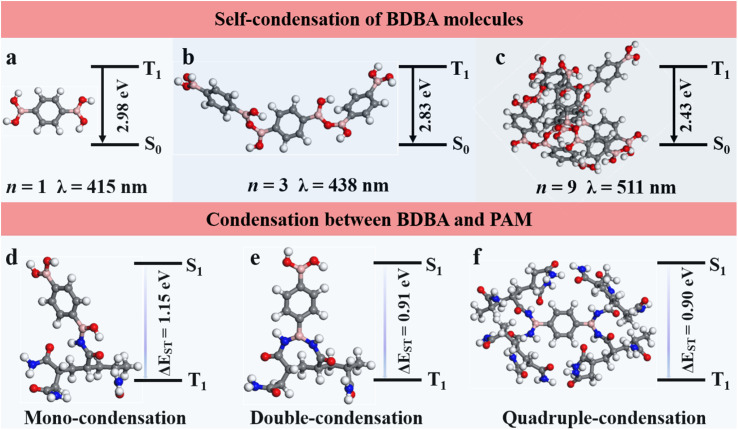
Structural models and the energy level diagrams between the triplet excited states and ground states for the (a–c) self-condensation of BDBA and (d–f) condensation between BDBA and PAM.

We explored time-dependent density-functional theory (TD-DFT) calculations to explain the mechanism of the good RTP and self-healing of the composites. For the RTP behavior studies, the T_1_ energy level of a singular BDBA molecule was calculated to be 2.98 eV, corresponding to the phosphorescence emission at 415 nm (Fig. 4a). However, the BDBA molecules are prone to aggregation and self-condensation, leading to a decrease in the T_1_ energy level and a red shift in the phosphorescence emission (Fig. 4b). The T_1_ energy level decreased to 2.43 eV when the number of self-condensed molecules was increased to 9, and the calculated wavelength of the theoretical phosphorescence emission red-shifted to 511 nm (Fig. 4c). These results indicated that the dark-blue phosphorescence of BDBA-PAM at 410 nm could be attributed to the mono-dispersed state of BDBA isolated by the polymer matrix.

The geometries of BDBA and BDBA-PAM were further explored. We studied the ability of the boronic acid groups in BDBA to condense with amide groups in PAM by calculating the free energy of condensation between BDBA and PAM. The results suggested that one boric acid group favored condensation with one amide group in a PAM chain with a free energy of 0.44 eV (Fig. 4d) rather than condensation with two neighboring amide groups in a PAM chain with an energy of 0.75 eV per condensation (Fig. 4e). Accordingly, the two boronic acid groups in a BDBA molecule were apt to condense with four different PAM chains to form a cross-linked network (Fig. 4f). The energy required for the hydrolysis of B–N bonds from the cross-linked films was calculated to be −0.44 eV per condensation, which could feasibly be realized. This result demonstrated the good self-healing capacities of the BDBA-PAM composite films. In comparison, poly(vinyl alcohol) (PVA) with hydroxyl groups was employed to react with the boric acid in BDBA through B–O bonds. The boronic acid in BDBA tended to condense with two neighboring hydroxyl groups in one PVA chain with a free energy of 0.11 eV per condensation, instead of condensing with a single hydroxyl group in the PVA chain with a free energy of 0.39 eV (Fig. S11). The energy required for hydrolysis in BDBA-PVA was estimated to be 0.28 eV, explaining its failure in self-healing.

Furthermore, BDBA-PVA showed the energy level difference from the triplet state to the singlet state to be 1.19 eV, which was larger than the energy level difference of 0.90 eV for BDBA-PAM. The smaller Δ*E*_ST_ value for BDBA-PAM suggested an easier intersystem crossing process and stronger RTP^40^ compared to BDBA-PVA. Accordingly, BDBA-PAM with B–N cross-linkage exhibited good RTP and self-healing performances.

### Versatility studies for self-healing RTP films

**Fig. 5:**
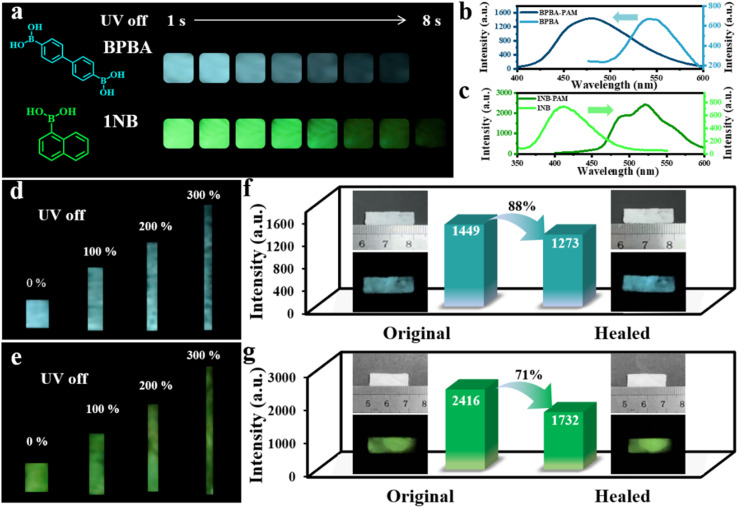
Phosphorescence and self-healing behaviors of BPBA-PAM and 1NB-PAM composite films. (a) Photos of the BPBA-PAM and 1NB-PAM composite films taken after UV light was turned off. Phosphorescence spectra of (b) BPBA powder (light blue), BPBA-PAM films (blue), (c) 1NB powder (light green), and 1NB-PAM films (green). Photos of the phosphorescence of the (d) BPBA-PAM and (e) 1NB-PAM composite films under different stretching rates. Phosphorescence recovery efficiencies of the (f) BPBA-PAM and (g) 1NB-PAM composite films after the healing process (insets show photos of the self-healing process under daylight and after UV light was turned off).

We further employed two other boronic acid-derived binders to investigate the versatility of B–N covalent bonding to prepare cross-linked self-healing composites with simultaneous recovery of RTP and mechanical properties. In brief, 4,4′-biphenyldiboronic acid (BPBA) and 1-naphthylboronic acid (1NB) were reacted with PAM to prepare BPBA-PAM and 1NB-PAM composite films. Bright phosphorescence emission could be observed after turning off the UV light, with a cyan emission of 7 s for BPBA-PAM and a green afterglow of 8 s for 1NB-PAM (Fig. 5a). In contrast, the phosphorescent afterglow of the BPBA and 1NB powder were hard to observe by the naked eye when the UV lamp was switched off (Fig. S12). The phosphorescence lifetime of 0.5 ms for BPBA powder was promoted to 710 ms for the BPBA-PAM composite, while the 1NB-PAM showed a longer lifetime of 695 ms compared to 0.2 ms for the 1NB powder (Fig. S13). Moreover, it was observed that phosphorescence emission of BPBA blue-shifted from 540 nm in the powder to 480 nm in the covalently cross-linked BPBA-PAM composite film (Fig. 5b). In contrast, the phosphorescence of 1NB was red-shifted from 410 nm in the powder to 490 nm and 520 nm for 1NB-PAM (Fig. 5c). These phenomena could be ascribed to the different molecular structures of the boronic-acid-modified chromophores. BPBA molecules with boronic acid groups attached at two ends of the biphenyl tend to form a cross-linked structure with PAM, and thus the BPBA was well localized by the PAM network. In contrast, 1NB with only one boronic acid could not be efficiently immobilized by PAM, leading to unavoidable molecular aggregation.

The mechanical and RTP properties under stretching for BPBA-PAM and 1NB-PAM composite films were also studied. Photographs of the phosphorescence were taken with stretching of the composite films to 0, 100, 200 and 300% of their original length, and bright cyan and green afterglows were observed throughout the BPBA-PAM and 1NB-PAM films (Fig. 5d and e).

The self-healing capacities of these composite films were then explored under the same conditions using water as the healing agent. With the addition of water along the fracture of the films, the cut pieces could be healed into one piece at room temperature within a few minutes. The phosphorescence intensity of BPBA-PAM recovered to 88% of the original value after the healing process (Fig. 5f), while the phosphorescence recovery of the 1NB-PAM film only reached 71% (Fig. 5g). Additionally, healing efficiencies of 90% and 75% were achieved for the mechanical properties of the BPBA-PAM and 1NB-PAM composite films, respectively (Fig. S14). Comprehensively comparing the materials, BDBA-PAM and BPBA-PAM possessed higher healing efficiencies in terms of both RTP and mechanical properties than 1NB-PAM (Fig. S15). This result could be ascribed to the greater possibility for BDBA and BPBA, which have two boronic acid groups at both ends of the benzene rings, to covalently bind to PAM to form a cross-linked network. Therefore, we have demonstrated the universality of the use of dynamic B–N bonding to prepare composite films with room-temperature self-healing and phosphorescence.

### Applications of self-healing RTP films

**Fig. 6:**
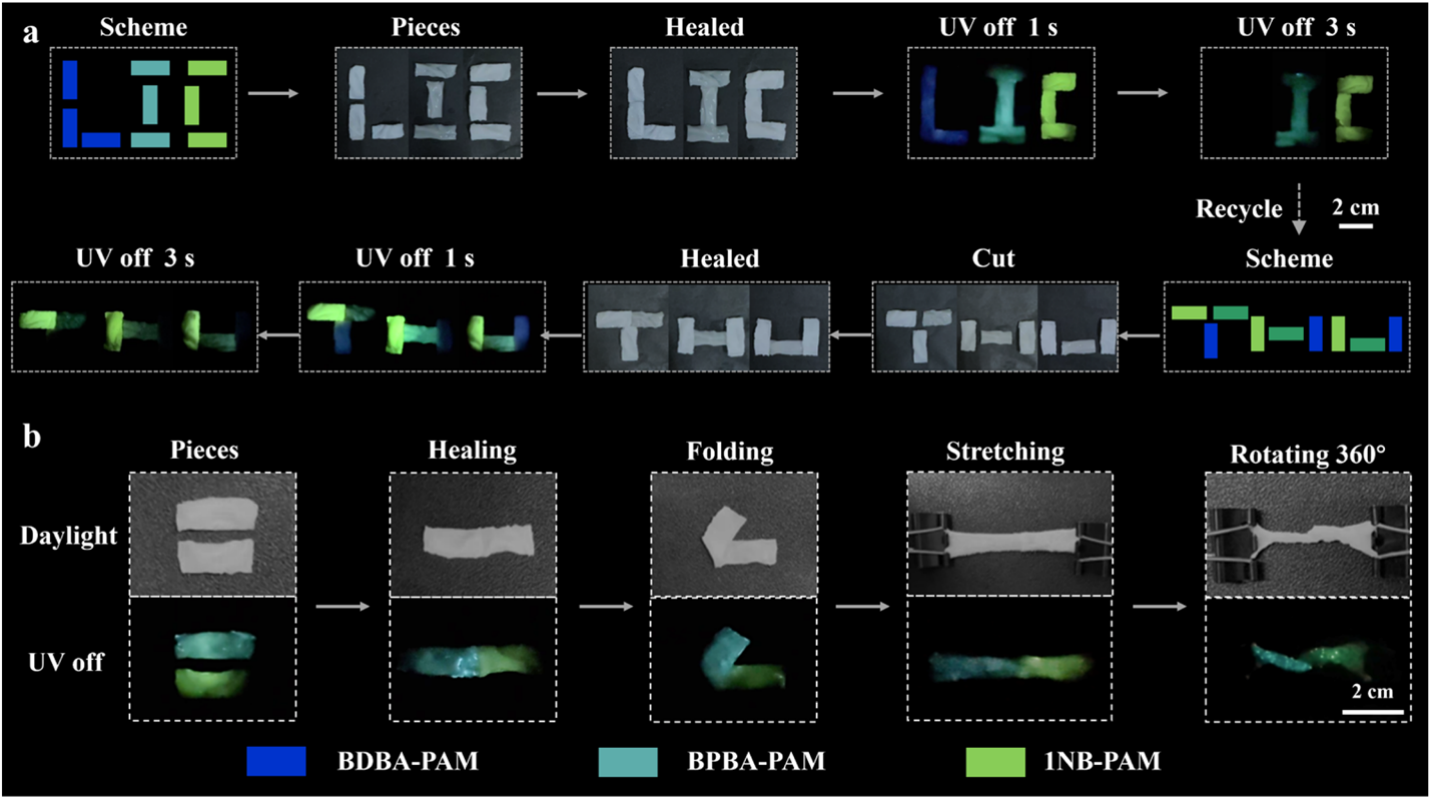
Recycling and anti-counterfeiting applications of the BDBA-PAM, BPBA-PAM and 1NB-PAM composite films. (a) Cutting and assembly into different letters and observation of their afterglows for anti-counterfeiting applications. (b) Folding, stretching and rotation of the healed composite films.

Anti-counterfeiting and recycling applications of the B–N based composite films were studied. As validated from the CIE coordinates, different phosphorescence colors ranging from blue-emissive BDBA-PAM (0.156, 0.083) to cyan-colored BPBA-PAM (0.156, 0.274) and greenish 1NB-PAM (0.218, 0.544) were identified and used for anti-counterfeiting (Fig. S16). First, BDBA-PAM, BPBA-PAM and 1NB-PAM composite films were cut into rectangles with a size of 2 × 1 cm^2^. The BDBA-PAM fragments were placed together to assemble the letter “L”, followed by the addition of water for a self-healing process (Fig. 6a). Similarly, the BPBA-PAM fragments were assembled into the letter “I”, and a letter “C” was assembled from the 1NB-PAM pieces. After healing in water for 5 min, their morphological completeness was recovered, and the phosphorescent pattern “LIC” was observed after the ultraviolet lamp was turned off. The assembled letters “L”, “I” and “C” showed dark-blue, cyan and green phosphorescence emission, respectively. The colored “LIC” pattern changed to “IC” after 3 s with the disappearance of the dark-blue emission of BDBA-PAM. In addition, the pattern composed of assembled BDBA-PAM, BPBA-PAM and 1NB-PAM fragments was cut and healed again for recycling *via* the reassembly process. Five minutes later, a “THU” pattern with integrated blue, cyan and green phosphorescence emissions was re-organized *via* healing with water. The composite films retained their RTP properties after the recycling process. After a delay of 3 s, an irregular pattern with cyan and green afterglows was displayed, demonstrating potential applications for multi-stage encryption.

In addition, we also explored the flexibility and deformability of the healed films. Pieces of BPBA-PAM and 1NB-PAM composite films with a rectangular shape were utilized for the tests. These films were healed with water and assembled into rectangles with cyan (left) and green (right) emission (Fig. 6b). The assembled composite films can be folded, stretched and rotated 360° without breaking at the healed site. Satisfactorily, the assembled composite films showed good mechanical properties and a bright dual-color phosphorescent afterglow throughout the whole film. These results highlighted the advantages of stretchable, self-healable and phosphorescent composite films based on dynamic B–N covalent bonds, which showed great potential to achieve encryption with long lifetime and lossless readout capabilities.

## Conclusions

In summary, we have proposed the use of B–N bonds between chromophore binders and the polymer matrix to establish a covalent cross-linked network for room-temperature self-healing composite films. The B–N bonds could construct a covalent network to increase the rigidity of the composite films for enhanced RTP performance, and the composite films showed good mechanical properties with uniform phosphorescence emission under stretching. More importantly, both the phosphorescence and mechanical properties could be simultaneously recovered for these covalently bonded films after several cutting–healing cycles. The proposed strategy is applicable to different binders with boronic acid groups, and the prepared composite films can be assembled and reassembled into a single piece after the self-healing process in water. These results highlight the power of dynamic covalent bonds in the structural optimization and performance enhancement of functional polymeric films. The proposed strategy shows good versatility and potential for anti-counterfeiting and information storage applications. It is expected that the proposed strategy could be further extended for the design and preparation of multi-functional materials.

## Author contributions

C. L., and R. T. conceived the experiments. Y. W. carried out the experiments. Y. Y. conducted theoretical calculations. Y. W., K. L., Y. Y., R. T., and C. L. contributed to data analysis and writing of this manuscript. Y. W., K. L., and Y. Y contributed equally to this work.

## Conflicts of interest

There are no conflicts to declare.

## Supplementary Material

SC-017-D5SC09282E-s001

## Data Availability

The data supporting this article have been included as part of the supplementary information (SI). Supplementary information: phosphorescence spectra and afterglow diagrams of BDBA molecules; energy dispersive spectrometer mapping and temperature-dependent phosphorescence lifetimes of BDBA-PAM films; mechanical property curve of PAM film; healing process for pure PAM, BDBA-PAM and BDBA-PVA films; phosphorescence spectra and stress–strain of BDBA-PAM films at different healing time; XPS analysis for BDBA, PAM and BDBA-PAM; theoretical calculation for the reaction between BDBA and PVA; healing performances and CIE coordinate charts for composite films prepared by different binders. See DOI: https://doi.org/10.1039/d5sc09282e.
