# Supplementary material for: Breaking the paradox: simultaneous recovery of phosphorescence and mechanical properties in polymeric films
Source: Chem Sci. 2025 Dec 29;17(9):4794–802. doi: 10.1039/d5sc09282e (PMC12809460; doi:10.1039/d5sc09282e)
Supplement: SC-017-D5SC09282E-s001 [file SC-017-D5SC09282E-s001.pdf]

Supplementary Information (SI) for Chemical Science.
This journal is © The Royal Society of Chemistry 2026

Supporting Information for

Break the paradox: simultaneous recovery of phosphorescence and mechanics for polymeric films

Yan Wang,†[a] Kaitao Li,†[a,c] Yongpeng Yang,†[b] Rui Tian,*[a,c] Chao Lu*[a,b,c]

[a]State Key Laboratory of Chemical Resource Engineering, Beijing University of Chemical Technology, Beijing, 100029, China

[b]Pingyuan Laboratory, College of Chemistry, Zhengzhou University, Zhengzhou, 450001, China

[c]Quzhou Institute for Innovation in Resource Chemical Engineering, Quzhou, 324000, China

†Yan Wang, Kaitao Li and Yongpeng Yang contributed equally to this work.

***Corresponding author**

E-mail: tianrui@mail.buct.edu.cn (R. Tian); luchao@mail.buct.edu.cn (C. Lu)

Table of Contents

Experimental Section S3
Figure S1. Excitation and emission spectra of BDBA S5
Figure S2. Photographs of the afterglow for BDBA molecules S6
Figure S3. Low temperature spectra of BDBA powder and in solution S7
Figure S4. Energy dispersive spectrometer mapping of BDBA-PAM films S8
Figure S5. Temperature-dependent phosphorescence lifetimes of 1% BDBA-PAM S9
Figure S6. Mechanical property curve of PAM film. S10
Figure S7. Photographs of the healing process for pure PAM and BDBA-PAM composite films. S11
Figure S8. Healing process and RTP behaviors of 1% BDBA-PVA films S12
Figure S9. Phosphorescence spectra and stress-strain of BDBA-PAM films at different healing time S13
Figure S10. XPS spectra of C 1s for BDBA, PAM and BDBA-PAM composite films before and after healing process S14
Figure S11. Structural models and energy level diagrams between the triplet excited and ground states of BDBA and PVA with different degrees of condensation S15
Figure S12. Photographs of the afterglows for BPBA and 1NB powder after turning off the UV lamp S16
Figure S13. Phosphorescence lifetime measurements for (a) BPBA powder and BPBA-PAM composite film, (b) 1NB powder and 1NB-PAM composite film S17
Figure S14. Mechanical curves of BPBA-PAM and 1NB-PAM before and after healing. S18
Figure S15. Self-healing efficiencies of RTP and mechanical properties for BDBA-PAM, BPBA-PAM and 1NB-PAM films. S19
Figure S16. CIE 1931 coordinate charts of BDBA-PAM, BPBA-PAM and 1NB-PAM composite films. S20
Table S1. RTP and strain efficiency values at different healing time in water S21
Table S2. RTP and strain efficiency values at different healing cycles in water S22
Table S3. XPS peak information of B 1s of PAM, BDBA, BDBA-PAM before and after healing process S23
Table S4. XPS peak information of N 1s of PAM, BDBA, BDBA-PAM before and after healing process S24
Table S5. XPS peak information of C 1s of PAM, BDBA, BDBA-PAM before and after healing process S25
References S26

Experimental Section

Materials. Analytical-grade chemicals, including K_2CO_3, $KHCO_3$ and dimethyl sulfoxide (DMSO) were purchased from Sinopharm Chemical Reagent Co. Ltd. (Shanghai, China). Polyacrylamide (PAM) and polyvinyl alcohol (PVA) were obtained from HWRK Co. Ltd. (Beijing, China). 1,4-Phenylenediboronic acid was purchased from J&K Scientific Ltd. (Beijing, China). 4,4'-Biphenyldiboronic acid (BPBA) and naphthalene-1-boronic acid (1NB) were acquired from Aladdin Chemical Co. Ltd. (Shanghai, China). All the chemicals used in this experiment were analytical reagent and used without further purification.

Preparation of *x*%BDBA-PAM and referenced films. PAM aqueous solution (5 wt%) was prepared by dissolving PAM (0.5 g) in deionized water (10 mL) under stirring at room temperature. A buffer solution of pH = 10.5 was prepared by K_2CO_3 and $KHCO_3$. BDBA (5 mg) was dissolved in a mixture of 4 mL DMSO and 1 mL buffer solution, and the hydrolyzed BDBA solution with the concentration of 1 mg/mL was obtained. The acquired BDBA solution was added to the PAM aqueous solution under continuous stirring for 7 hours at room temperature, followed by the drying in an oven at 60 °C for 10 hours. Finally, the composite films were acquired and labelled as *x*% BDBA-PAM (*x*% represented the weight ratio of BDBA to PAM, which varied in the ranges from 0.8 wt%, 1 wt%, 1.2 wt %, to 1.5 wt %). As a reference, pure PAM (5 wt%), 1% BDBA-PVA, 1% BPBA-PAM, and 1% 1NB-PAM films were prepared according to the same procedures.

Similarly, pure PAM film was prepared by drying PAM aqueous solution (5 wt%) at 60 °C for 3 h.

Self-healing experiments for the films. The prepared composite films were cut into two pieces with scissors, and the healing process was proceeded by adding water along the fracture of the films. Afterwards, these films were left at the room temperature for 0 – 14 h for the healing process. These procedures were implemented repeatedly for different cycles to explore the healing efficiency for the composite films. The phosphorescent and mechanical properties for the healed films were studied under different healing cycles.

Sample characterization. X-ray diffraction patterns (XRD) of all the films were measured on an Ultima IV diffractometer in the range from 3° to 90°. Fourier transform infrared spectroscopy (FT-IR) spectra of the films were measured on a Nicolet 6700 in the range of 500 – 4000 cm^{-1}. UV-vis absorption spectra of samples were measured on a Shimadzu UV-3600 spectrophotometer (Japan). The phosphorescence photos for the composite films were captured after irradiation under a 280 nm UV LED module for 10 s. The phosphorescence spectra of all the samples were recorded using an F-7000 spectrophotometer (Hitachi, Japan) with an excitation wavelength of 280 nm. The phosphorescence spectra were collected in the range of 300 – 600 nm with the voltage of 800 V. CIE 1931 coordinate charts for the composite films were acquired based on the phosphorescence spectra. Phosphorescence lifetime measurements and temperature-dependent lifetimes were implemented on an Edinburgh Instruments FLS 980 fluorimeter. Leica TCS SP8 Confocal laser scanning microscope (CLSM) was used to monitor the self-healing process of the films in the bright field using a 10x lens. The tensile strengths of the as-prepared films were measured using a testing machine CMT4104 (MTS, China) with an extension rate of 10 mm/min. X-ray photoelectron spectroscopy (XPS) measurements were carried out on ESCALAB 250 instrument (Thermo Electron) with Al Kα radiation. The scanning electron microscope (SEM) images, energy dispersive spectrometer (EDS) analysis and mapping of the samples were captured on S-4700 (Hitachi, cold field emission).

Theoretical simulation of chromophore molecules and composite materials. In the theoretical simulations, the energies of the individual energy levels were calculated after a thorough optimization of geometries for the chromophore themselves and the polymeric composites using the M06-2X hybrid functional and the 6-31G* basis set (The M06 suite of density functionals for main group thermochemistry, thermochemical kinetics, noncovalent interactions, excited states, and transition elements: two new functionals and systematic testing of four M06-class functionals and 12 other functionals). The energy levels of excited states for the chromophores and composites were further evaluated using the TD-m062x/6-31g* with Gaussian 16 program, and the phosphorescence energy calculations were performed as vertical emission energy calculations.[1,2]

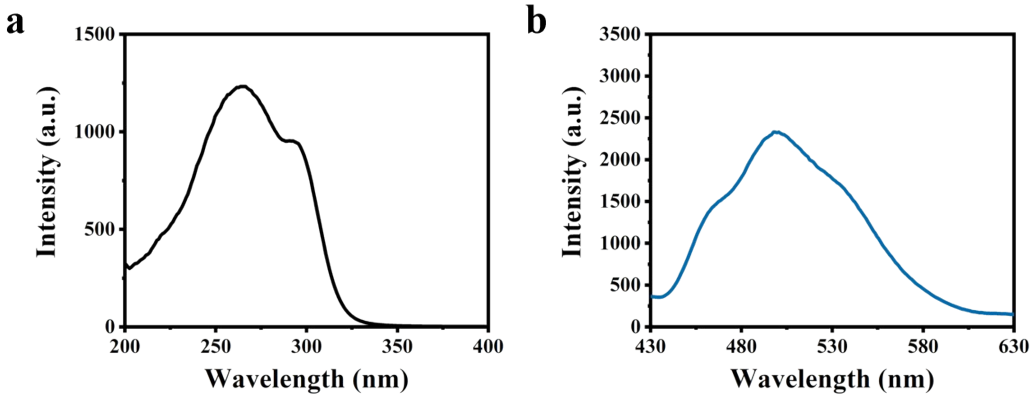

Fig. S1 (a) Excitation and (b) emission spectra of 1,4-benzenediboronic acid (BDBA).

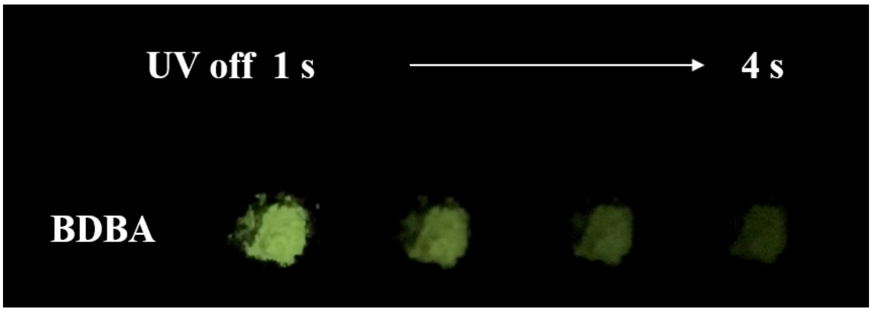

Fig. S2 Photographs of the afterglow for BDBA molecules after tuning off the UV lamp.

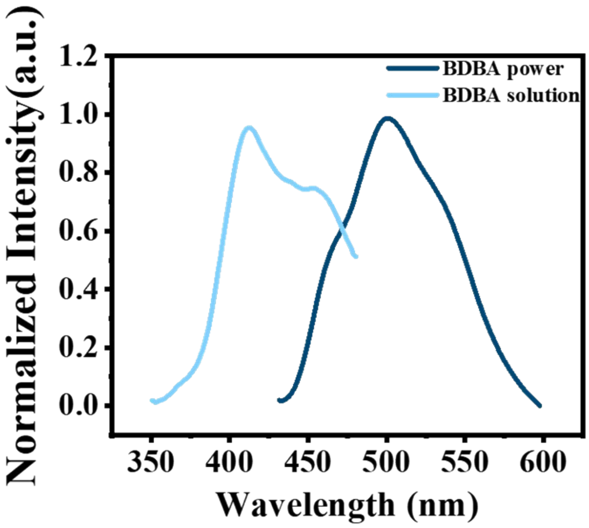

Fig. S3 Low temperature spectra of BDBA powder and solution recorded in liquid nitrogen.

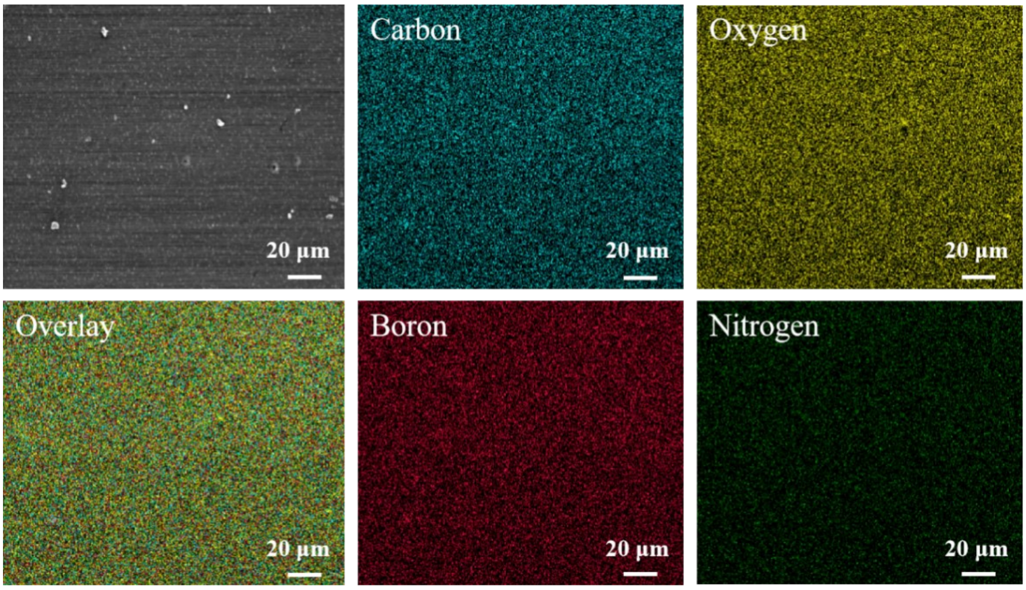

Fig. S4 Energy dispersive spectrometer mapping of carbon, oxygen, boron and nitrogen elements for BDBA-PAM films.

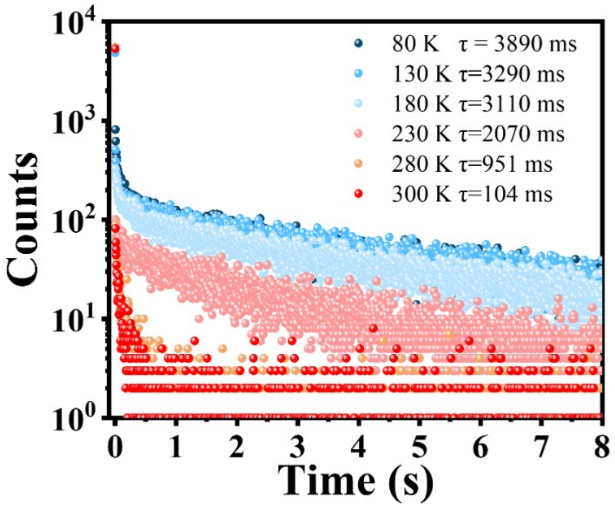

Fig. S5 Temperature-dependent phosphorescence lifetimes of 1.0% BDBA-PAM recorded from 80 to 300 K.

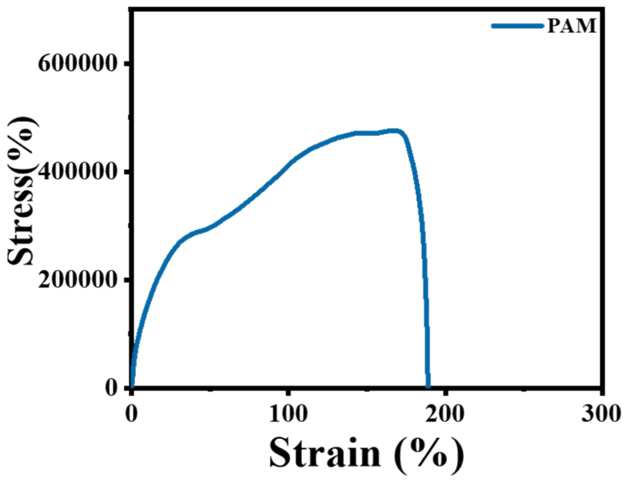

Fig. S6 Mechanical property curve of PAM film.

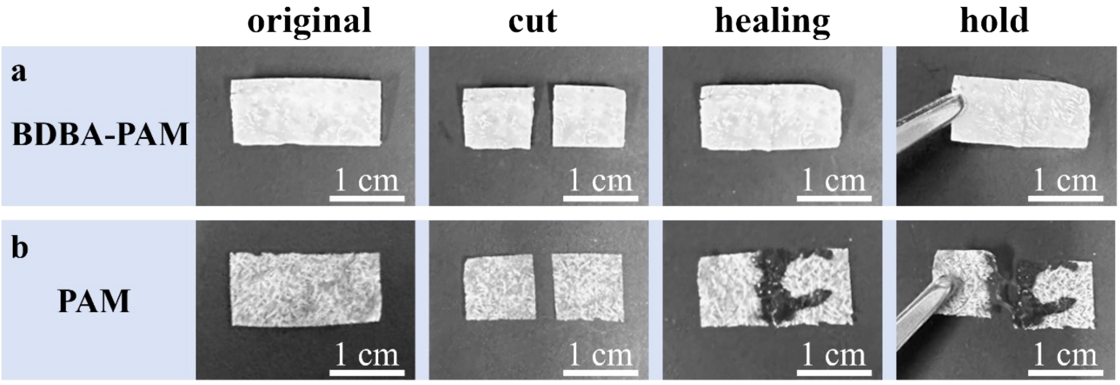

Fig. S7 Photographs of the healing process for (a) BDBA-PAM and (b) pure PAM films.

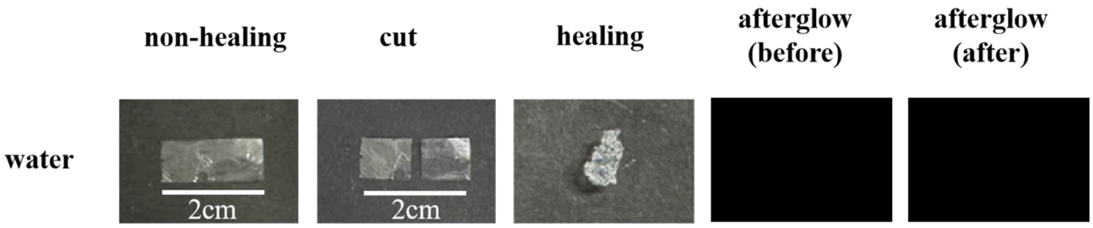

Fig. S8 Healing process and RTP behaviors of 1% BDBA-PVA films.

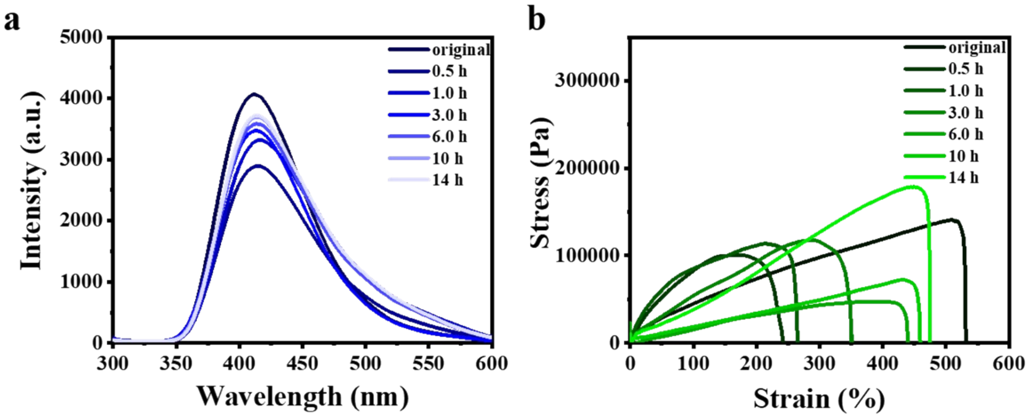

Fig. S9 (a) Phosphorescence spectra and (b) stress-strain data of BDBA-PAM films under different healing time.

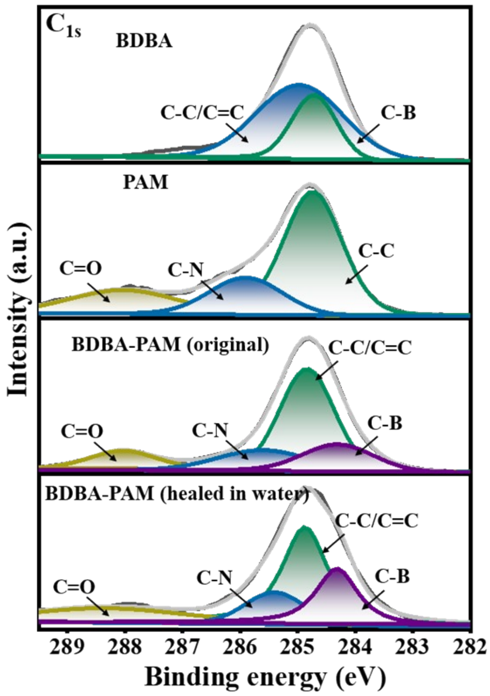

Fig. S10 X-ray photoelectron spectroscopy (XPS) spectra of C 1s for BDBA, PAM and BDBA-PAM composite films before and after healing process.

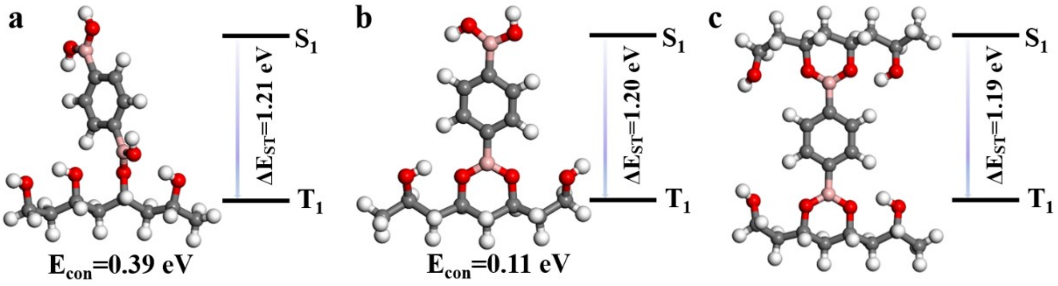

Fig. S11 Structural models and energy level diagrams between the triplet excited and ground states for (a) monocondensation, (b) double condensation, (c) quadruple condensation of BDBA and PVA.

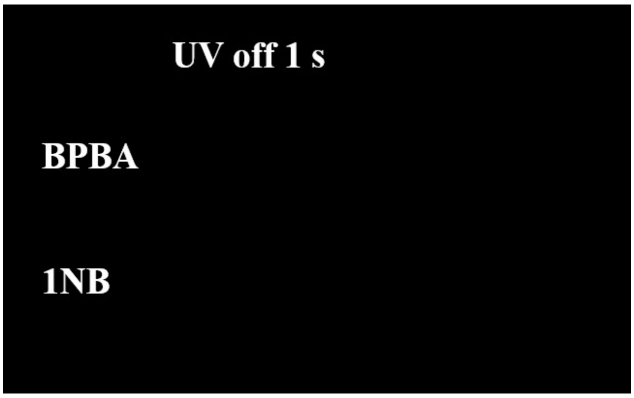

Fig. S12 Photographs of the afterglows for BPBA and 1NB powder after turning off the UV lamp.

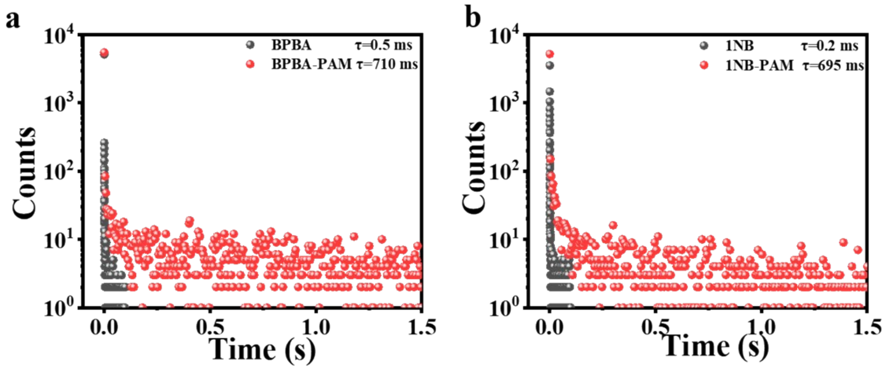

Fig. S13 Phosphorescence lifetime measurements for (a) BPBA powder and BPBA-PAM composite film, (b) 1NB powder and 1NB-PAM composite film.

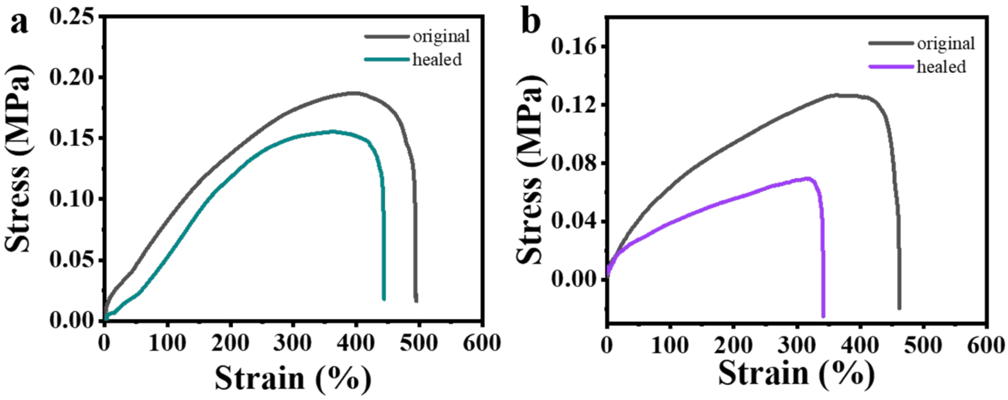

Fig. S14 Mechanical curves of (a) BPBA-PAM and (b) 1NB-PAM before and after healing.

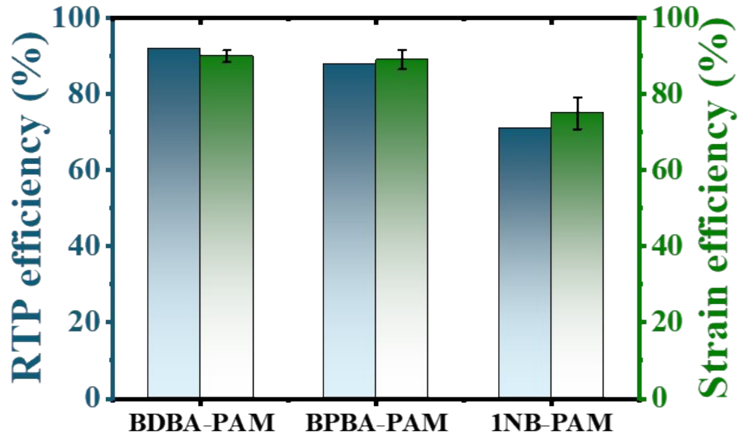

Fig. S15 Self-healing efficiencies of RTP and mechanical properties for BDBA-PAM, BPBA-PAM and 1NB-PAM films, respectively.

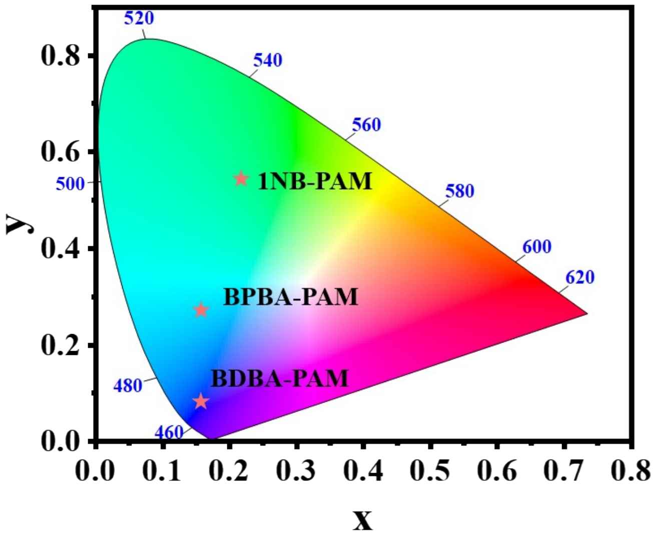

Fig. S16 CIE 1931 coordinate charts of BDBA-PAM, BPBA-PAM and 1NB-PAM composite films.

Table S1 RTP and strain efficiency values for BDBA-PAM films at different healing time in water.

Healing time (h)	RTP efficiency (%)	Strain efficiency (%)
**0.5**	71	45
**1**	81	49
**3**	85	65
**6**	88	81
**10**	91	86
**14**	92	90

Table S2 RTP and strain efficiency values for BDBA-PAM films at different healing cycles in water.

Cycle	RTP efficiency (%)	Strain efficiency (%)
**1**	96	91
**2**	93	86
**3**	90	83

Table S3 XPS peak information of B 1s of PAM, BDBA, BDBA-PAM before and after healing process.

	**B-$(OH)_2$**	**B-C**	**B-N**
**PAM**	–	–	–
**BDBA**	50.0%	50.0%	–
**BDBA-PAM (original)**	16.2%	45.2%	38.6%
**BDBA-PAM (healed)**	21.1%	41.8%	37.1%

Table S4 XPS peak information of N 1s of PAM, BDBA, BDBA-PAM before and after healing process.

	N-C	N-H	N-B
**PAM**	33.3%	66.7%	–
**BDBA**	–	–	–
**BDBA-PAM (original)**	33.2%	33.5%	33.3%
**BDBA-PAM (healed)**	34.1%	33.2%	32.7%

Table S5 XPS peak information of C 1s of PAM, BDBA, BDBA-PAM before and after healing process.

	**C-C/C=C**	**C-N**	**C-B**	**C=O**
**PAM**	59.8%	20.1%	–	20.1
**BDBA**	69.3%	–	30.7	–
**BDBA-PAM (original)**	51.9%	16.2%	17.5%	14.4%
**BDBA-PAM (healed)**	47.5%	13.2%	21.2%	18.2%

References:

1 Gaussian 16, Revision A.03, M. J. Frisch, G. W. Trucks, H. B. Schlegel, G. E. Scuseria, M. A. Robb, J. R. Cheeseman, G. Scalmani, V. Barone, G. A. Petersson, H. Nakatsuji, X. Li, M. Caricato, A. V. Marenich, J. Bloino, B. G. Janesko, R. Gomperts, B. Mennucci, H. P. Hratchian, J. V. Ortiz, A. F. Izmaylov, J. L. Sonnenberg, D. Williams-Young, F. Ding, F. Lipparini, F. Egidi, J. Goings, B. Peng, A. Petrone, T. Henderson, D. Ranasinghe, V. G. Zakrzewski, J. Gao, N. Rega, G. Zheng, W. Liang, M. Hada, M. Ehara, K. Toyota, R. Fukuda, J. Hasegawa, M. Ishida, T. Nakajima, Y. Honda, O. Kitao, H. Nakai, T. Vreven, K. Throssell, J. A. Montgomery, Jr., J. E. Peralta, F. Ogliaro, M. J. Bearpark, J. J. Heyd, E. N. Brothers, K. N. Kudin, V. N. Staroverov, T. A. Keith, R. Kobayashi, J. Normand, K. Raghavachari, A. P. Rendell, J. C. Burant, S. S. Iyengar, J. Tomasi, M. Cossi, J. M. Millam, M. Klene, C. Adamo, R. Cammi, J. W. Ochterski, R. L. Martin, K. Morokuma, O. Farkas, J. B. Foresman, and D. J. Fox, Gaussian, Inc., *Wallingford CT*, 2016.

2 Y. Zhao, D. G. Truhlar, *Theor. Chem. Acc.*, 2008, **120**, 215-241.
